# Language-games in live mindfulness-based stress reduction: a philosophy of language analysis of participant-trainer dialogue

**DOI:** 10.3389/fpsyg.2026.1660807

**Published:** 2026-03-17

**Authors:** Ingeborg van den Bold, Sanneke de Haan, Jenny Slatman

**Affiliations:** Department of Culture Studies, Tilburg School of Humanities and Digital Sciences, Tilburg University, Tilburg, Netherlands

**Keywords:** Austin, body awareness, language-game, MBSR, mindfulness, philosophy of language, speech act, Wittgenstein

## Abstract

**Introduction:**

It is important to explore how words are given to body awareness in Mindfulness-Based Stress Reduction (MBSR), as this impacts health and illness, while the literature on this topic is scarce. This study is the first to explore the learning process of enhancing one's body awareness live in MBSR sessions through a philosophy of language lens. It is the first known application of Wittgenstein and Austin to full-course, live MBSR dialogue, and it analyzes language-games and all three speech acts in context. This is a suitable approach, as these philosophers focus on how language is used in real-life conversations.

**Methods:**

We analyzed the full transcript of a complete MBSR training with interpretative phenomenological analysis.

**Results:**

The results indicate that verbalizing body awareness was difficult for MBSR participants. Participants talked about emotions or they made rational judgments, while they found it hard to express what bodily sensations they felt. We suggest, using Wittgenstein's concept of “language-games,” that in this case study, learning to verbalize one's body awareness can be understood as learning a language-game of “reporting sense perceptions.” Referring to Austin's concept, our findings also show what type of “speech acts” are done in MBSR.

**Discussion:**

The results of this study align with insights on emotion regulation therapy. The first step in these therapies is learning to feel bodily sensations instead of making rational judgments. In conclusion, we suggest that our results contribute to the scientific debate on the relation between language and body awareness. We also hypothesize the implications for the widespread use of mindfulness apps and recorded versions of the body scan, that both lack the feedback of the living trainer-participant dialogue.

## 1 Introduction

Body awareness is about paying attention to sensations in one's own body and being able to feel them (Ginzburg et al., 2014). Body awareness is considered to be an important element for the effects of mindfulness-based interventions (Shusterman, 2008; Hölzel et al., 2011; Mehling et al., 2011; Gotink et al., 2017). One of the well-studied interventions is Mindfulness-Based Stress Reduction (MBSR) (Baer, 2003; Santorelli and Kabat-Zinn, 2014). MBSR is originally rooted in Buddhist meditative traditions and is the form in which Kabat-Zinn introduced mindfulness to Western healthcare (Kabat-Zinn, 1982, 2013). MBSR is a standardized mindfulness group training, without the Buddhist context and terminology. It includes formal practices such as body scan meditation, sitting meditation, walking meditation, and mindful hatha yoga. In MBSR, yoga postures are practiced as an embodied form of meditation, just as the body scan or the walking meditation. The emphasis in this yoga-informed mindful movement is therefore not on physical exercise but on moment-to-moment awareness of the body (Santorelli and Kabat-Zinn, 2014; Kabat-Zinn, 2020, p. 100–101). “Mindfulness” is a multivariate concept that not only can be understood as referring to an intervention strategy but also to a process, or to a skill (Munoz-Martinez et al., 2017). Moreover, mindfulness is conceptualized in various ways in different speech communities (Stofleth and Manusov, 2019; Boxer et al., 2025). MBSR, however, draws on Kabat-Zinn's (2020) conceptualization of mindfulness as “the awareness that arises by paying attention on purpose, in the present moment, and non-judgmentally” (p. xxxvii). This awareness is embodied. By cultivating this embodied awareness, MBSR aims at increasing “our capacity to embrace the actuality of things” (p. xxviii), which, according to Kabat-Zinn, is healing and transforming. Aimed benefits include improved relationships with ourselves and with others, stress reduction, improved health, wellbeing, and happiness, and living a meaningful life. Studies show evidence for the benefits of mindfulness interventions for patients with depression (Goldberg et al., 2018; Nandarathana and Ranjan, 2024), pain conditions and addiction (Goldberg et al., 2018), and for stroke patients (Hisan et al., 2025). Benefits have also been shown for patients with cancer (Ledesma and Kumano, 2009; Menon and Saranya, 2025; Komariah et al., 2025; Wang et al., 2025), cardiovascular disease (Scott-Sheldon et al., 2020), and anxiety disorders (Hoge et al., 2023; Nandarathana and Ranjan, 2024; Bhattacharya and Yadav, 2025). The value of mindfulness interventions has also been established in studies on individuals in good health (Khoury et al., 2015; Cardenas et al., 2025). Mindfulness as an intervention strategy is increasingly used in therapeutic settings (Van Dam et al., 2018). It is also applied outside medical recommendations in organizational contexts (Esteves et al., 2025). It is used to become more effective and productive in work (Mordue, 2021; Esteves et al., 2025) and to prevent burnout (Knudsen et al., 2023; Esteves et al., 2025). In organizational contexts, mindfulness interventions are also used to increase leadership and management competencies (Esteves et al., 2025). The use of mindfulness interventions to increase productivity in work has also been critically referred to as “McMindfulness” (Purser, 2019).

While body awareness is considered important in mindfulness practices, in medical contexts, it can be adaptive or maladaptive: body awareness can alleviate or reinforce illness experience (White et al., 2010; Ginzburg et al., 2014; Lööf et al., 2014; Mehling, 2020; Puranen, 2022). Several mechanisms have been described for these different effects. For instance, a negative feedback loop may occur in cardiac disease patients: hypervigilant body awareness induces anxiety, which generates bodily signals that are interpreted as disease symptoms, which reinforces anxiety (White et al., 2010). Another feedback loop that has been described is between pain awareness and maladaptive pain behavior: reducing physical activity because of pain leads to increased pain in the long term (Naye et al., 2021). Positive or negative effects of body awareness are also related to the way one gives meaning to bodily sensations in words (Dreeben et al., 2013; Chiffi et al., 2021). Catastrophizing interpretations of bodily signals in words can have a negative, nocebo effect, whereas positive interpretations can have a positive, placebo effect (Darnall and Colloca, 2018). Since it matters how one gives words to bodily sensations in medical contexts, how words are used in the process of enhancing one's body awareness in mindfulness training is also relevant.

Empirical research on language use in the learning process of enhancing one's body awareness in MBSR is scarce, as we will show in the next subsection. This study addresses this research gap. This study is the first to explore how language is used in the learning process of enhancing one's body awareness live in MBSR sessions through a philosophy of language lens. It draws on the philosophy of language developed by Wittgenstein and by Austin. This may be a suitable approach, as these philosophers specifically focus on how language is used in real-life conversations.

### 1.1 Language and body awareness

In studies on the role of language in the process of enhancing one's body awareness, conflicting positions can be discerned (Table 1). In some contemplative traditions, language is said to stand in the way of experiencing one's body and being “an obstacle to deepening the practice” (Petitmengin et al., 2019, p. 56). Others, for instance, (Krägeloh 2019), say that giving words to body awareness is not possible at all, as this awareness is non-conceptual and therefore in principle cannot be expressed in language. Yet others claim that giving words to body awareness is not necessary for body awareness and should not be considered a good measure for body awareness either. For instance, according to Zen Buddhists interviewed by Christopher et al. (2014), there is a difference between being aware of how one feels and the ability to describe those feelings. Being good with words is not the same as being aware. By contrast, yet others argue that language can increase body awareness, in spite of the intricacies involved. Shusterman (2008), for instance, argues that, although language can be too limited for verbalizing what one feels, “linguistic tags or descriptions” (164) can help to discriminate what one feels. These linguistic tags may even transform bodily feelings.

**Table 1:** Different perspectives on the relation between language and body awareness.

**The relation between language and body awareness**	**References**
Language is a hindrance to body awareness	Petitmengin et al., 2019
Language cannot express body awareness	Krägeloh, 2019
Language is not necessary for body awareness	Christopher et al., 2014
Language can increase body awareness	Shusterman, 2008

Empirical research on the use of language in the learning practice of enhancing one's body awareness in mindfulness training is rather scarce. There has been some qualitative research exploring language use in meditative, potentially body awareness-enhancing practices. In an autoethnographic study on walking meditation, which is also practiced in MBSR, Ball (2015) described how linguistic instructions are necessary to become aware of the body. However, at the same time, “descriptions fail to reveal the fluid nature of the doing of the practical embodied activity” (399). In another study, based on qualitative interviews with three mindfulness trainers, Schuling (2012) found that these trainers avoided the use of personal pronouns; they talked about “breathing” instead of “your breath” (7). These findings concord with Mamberg et al. (2015), who analyzed a recorded version of the body scan (an exercise in MBSR to enhance one's body awareness). They found that bodily sensations were noted “impersonally, rather than possessed” (1). These findings align with Nutall (2024), who performed an analysis of a mindfulness app, and observed that “the” was frequently used to indicate body parts or bodily sensations, for instance “the feet” or “the breath” (10).

Empirical studies on the use of language in MBSR or other meditative practices that apply the philosophy of language to understand their study results are even more scarce. One of these studies was done by Petitmengin et al. (2017), 2019). According to the participants they interviewed, words did not express exactly their experiences of body awareness. However, all participants agreed that making the effort of finding the right words sharpened their awareness of what they experienced in their bodies (Petitmengin et al., 2019). Petitmengin et al. (2017) mentioned Austin's notion of perlocutionary effect—the effect that the speaker's words have on the listener—to understand that the interviewer's questions sharpened participants' body awareness. Another study that applies the philosophy of language is Mathews and Anderson's (2021) interview study. They found that some participants in MBSR struggled with the specific mindfulness language used in the course. For some participants, certain terms used in the instructions—terms not further specified by the authors—were too unfamiliar. Moreover, some participants found the way instructions were formulated as invitations too soft, regarding them as “sugar-coated” and “New-Ageist” (1,217). Others, by contrast, found this invitational language helpful. Mathews and Anderson referred to Wittgenstein to interpret their result that participants struggled with specific mindfulness language: learning to speak the language is an integral part of learning a practice.

None of the aforementioned studies analyzed the use of language in the learning practice of enhancing one's body awareness in the actual live situation of an MBSR training course. This is what we did in this study. This study is, to our knowledge, the first analysis of live training dialogue through a philosophy of language lens. The relation between language and body awareness is complicated, and words play an important role in the teaching setting. Therefore, it is important to explore how language is actually used in the learning process of enhancing one's body awareness during mindfulness training sessions.

### 1.2 Study purpose

This study addressed the following research questions: How do participants in an 8-week mindfulness training verbalize their experiences of body awareness? How are MBSR participants stimulated by the trainer to give words to their bodily experiences? How is language used by the MBSR trainer to enhance participants' body awareness? To address these questions, we used a theoretical framework built on concepts of the philosophy of language as developed by Wittgenstein and Austin. By means of this language philosophical framework, we were able to investigate how language was actually used in live MBSR sessions. This method differed from the *post-hoc* reflection of interview studies. This method also differed from linguistic methods such as word counting, documenting conversational turn-taking, or analyzing the use of different verb forms. This language philosophical method allowed us to investigate what verbalizing body awareness actually means in the live MBSR trainer-participant dialogue.

## 2 Theoretical framework—Ordinary language philosophy

The research questions that we address in this study relate to one of the major questions asked in ordinary language philosophy: “How to do things with words?” The epithet “ordinary” has been used to indicate that ordinary language philosophers, such as Wittgenstein and Austin, focus on the actual use of language in everyday conversations. This could make this branch of philosophy particularly apt for our analysis, as we wanted to know how language is actually used in the practice of an MBSR course.

What a word means is how it is *used*, Wittgenstein (1953/2009) argues in *Philosophical Investigations*. Language is used in very many ways: language is used in different “language-games” (14–15). Wittgenstein gives all sorts of examples of language-games. He explains his use of the word “language-game” to emphasize “that *speaking* the language is part of an activity, or a form of life” (15, emphasis in original). Rules of the game can be taught, or someone may just learn the rules by watching other people play. Wittgenstein elaborates that he deliberately does not give a definition of “language-game,” for language-games do not have one thing in common that could help provide a definition. Like members of a family, games look alike in many different ways without having a single set of shared properties. Giving examples of different kinds of plays, “pointing” instead of giving a general definition, or “drawing any boundary” (38) is just the way we explain what a game is.

In *How to Do Things with Words*, Austin (1962) emphasizes—like Wittgenstein (1953/2009)—that we have to investigate how we actually make use of language in daily life. Then we will be able to understand the kind of things we are doing with words. Doing “constative” utterings—making statements that can be either true or false—is just one of the many ways in which language can be used (1–3). In ordinary language, we not only make constative statements, but we can, for instance, also ask questions or give orders. Therefore, it is not only what is being said, but also what we are doing while speaking, that matters. This doing creates something new that was not there before: speaking is “performative” (6)—it acts. To indicate that speaking is a form of doing, a way of performing, Austin introduces the concept of “speech act” (52). He distinguishes three types of speech acts: the *locutionary* act, the *illocutionary* act, and the *perlocutionary* act. The locutionary act is the act of uttering words and sounds while speaking. The illocutionary act is what the speaker is actually doing with this uttering of sounds. For instance, words can be used to make a promise, to give advice, or to express annoyance. Saying “I warn you” can imply a warning, advice, or a threat. The perlocutionary act refers to “what we bring about or achieve *by* saying something, such as convincing, persuading, deterring, and even, say, surprising or misleading” (109, emphasis in original). This perlocutionary effect does not merely depend on the words spoken but involves the whole speech act situation, including the relation between speaker and listener.

Therefore, both Wittgenstein and Austin emphasize that to understand the meaning of language, we have to look at how it is used. To analyze our data, we used their concepts of *language-games* and *speech acts*. This permitted us to analyze what is actually done with words in the process of enhancing one's body awareness in MBSR.

## 3 Methods

### 3.1 Study design, reflexivity statement, participants

This study is based upon a qualitative research methodology informed by a language philosophical theoretical framework. Using participant observation for data collection, we stayed close to the participants' perspective. Drawing on Interpretative Phenomenological Analysis to analyze the transcript, we stayed close to the words participants used themselves to describe their experiences. Interpreting these data through the theoretical frame of language philosophy, we stayed close to how language was actually used. As we will elaborate on in the next paragraphs, this three-layered methodology was particularly apt to study how language was used in the process of enhancing one's body awareness in MBSR.

This study was designed as a participant observation of a complete MBSR training. In participant observation, the researcher is embedded in a situation to understand the participants' perspective. The division between participation and observation varies per study (Conrad, 2001). In this study, the participating first author took fieldnotes and audio-recorded the whole training, which together formed the observation part of the study. During the MBSR training sessions, the participant author fully participated in the MBSR training, just like the other participants, which constituted the participating part of the study. Inherent to participant observation is that the participant researcher has a dual role: as a participant and as an observer. The participant researcher made an effort to disturb the MBSR sessions by her presence as little as possible and to prevent bias by her presence as much as possible. She made field notes in the same way as other participants also took notes for themselves. Additionally, the audio recording of the sessions was done in an inconspicuous, non-disturbing way. At the end of the last training session, a debriefing round was done by the participant researcher, in which the participants spontaneously said they had experienced the first author as a full fellow member of the group, and that the first author had been equally open about her own MBSR process as the other members. All three authors took good care to prevent bias as much as possible in interpreting the data. This, however, was not so different from preventing bias in research with a non-participating methodology: recognizing and bracketing prejudices. Preventing bias in the analysis part of the study was an intensive self-reflective process for the first author. To facilitate this self-reflective process, the first author also journaled about her emotions and feelings that arose during the analysis.

The number of participants in this study was 12. This number consisted of 11 MBSR participants, including the participating first author, plus the MBSR trainer (4 men, 8 women, age range 24–52 years old, all European participants). Most participants lived in an urban area (in three different cities in the South of the Netherlands). Some participants lived in rural villages (two different villages in the South of the Netherlands). The education level of the participants was mixed; however, most participants were theoretically educated. Nearly all participants had a job outside their own household, although some of them had been on longer sick leave. Marital status was mixed: some were married, some were unmarried, and some had been divorced. The majority of the participants had children. None of the participants had prior mindfulness experience, with the exception of the trainer. All participants were fluent in Dutch. All participants, including the trainer, signed informed consent to participate in the study. Participants were not compensated for their participation in the study. Ethical clearance was obtained from the research ethics board of Tilburg University (identification code: REDC # 2019/08).

### 3.2 Data collection

We conducted this research at a private mindfulness training institute in an urban area in the South of the Netherlands, from March until May 2019. Data were collected during all 8 training sessions of the MBSR training, varying in length from 2 $\frac{1}{4}$ to 2 $\frac{3}{4}$ h. Table 2 contains a session-by-session outline of the formal and informal training elements of the full MBSR program investigated in this study. The first author participated in the MBSR training and recorded all eight sessions, which were entirely spoken in Dutch. In order not to interfere with the natural discourse in the training, we did not do interviews. This is the reason that we did not collect further background information from the participants, other than what was said spontaneously during the MBSR sessions. The first author transcribed the recordings verbatim. Pseudonyms were used for individuals and for geographical names to protect participants' anonymity. These transcripts formed the data for analysis.

**Table 2:** Training elements per session in the MBSR program that was investigated in the present study.

**Training elements**	**1st session**	**2nd session**	**3rd session**	**4th session**	**5th session**	**6th session**	**7th session**	**8th session**
Body scan meditation	+	+		+	+	+	+	+
Sitting meditation	+	+	+	+	+	+	+	+
Walking meditation				+				
Yoga meditation			+					
Awareness of pleasant events		+	+			+	+	+
Awareness of unpleasant events	+	+	+	+	+	+	+	+
Mindfully doing daily activities			+			+	+	+
Mindful eating	+	+						+
Awareness of repetitive thoughts, emotions, and bodily sensations	+	+	+	+	+	+	+	+
Group conversations and dialogue	+	+	+	+	+	+	+	+
Guided meditation to evoke (un)pleasant feelings		+		+	+	+	+	+
Teacher instruction on mindfulness theory and concepts	+	+	+	+	+	+	+	+
Teacher instruction on stress theory	+	+	+	+	+	+	+	+

### 3.3 Data analysis

The transcripts—covering all that was said by the trainer and the participants during the MBSR course—provided a large and rich dataset (179,512 words). We analyzed the transcripts in line with interpretative phenomenological analysis (IPA). IPA is a method for qualitative research that looks at how different people make sense of a phenomenon in a given context. Instead of focusing on the content of particular experiences, IPA looks primarily at how these experiences are put into words. We considered IPA a suitable qualitative method for analyzing our transcripts. Indeed, IPA intends to explore experiences in the words that people use themselves, instead of arranging these experiences into predetermined categories (Smith et al., 2009). The purpose of this study was precisely to find out how people verbalize body awareness. Therefore, it was crucial to avoid prefixed categories, as this would have prevented us from finding out the words that people used themselves. In this study, the use of IPA can be recognized in the small number of participants and the use of long excerpts in the results section. The use of the method of IPA can also be recognized in the detailed analysis of the excerpts within the results section. For reasons of readability and coherence of this study, the detailed analysis of the excerpts could not be separated from the excerpts themselves. However, we preserved the broader reflection on our data and the discussion of our results for the discussion section.

After reading and rereading the data, the first author coded emerging keywords and themes throughout the whole transcript, using the open coding software (ATLAS.ti Scientific Software development GmbH, Berlin, Germany). This phase of the coding process was inductive—not led by theory but by the data. To ensure reliability, codes were verified by the third author, and parts of the transcript were also coded by the third author. The first and third authors compared their coding, and in an iterative deliberative process, a consensus was reached.

The next phase in this analysis was deductive—informed by our research questions regarding verbalizing body awareness. In this phase, all three authors were involved. First apart, and then together, we looked for overarching themes. These themes were all relevant to our research questions, as they were informed by them.

The last phase of our analysis was also deductive—informed by the philosophy of language discussed in our theoretical framework. The three authors together reflected on the overarching themes in the analysis, and we looked at how we could bring them down to just a few main themes.

After having completed the inductive and deductive phases of our analysis, we selected fragments from the transcripts to present in the results section. We translated the selected fragments from Dutch to English, doing justice to the stuttering and faltering parts, as our focus in this study was precisely on the use of language.

## 4 Results

### 4.1 Overview of the results

The first phase of our data analysis resulted in 120 descriptive codes. Examples show their variety: “Experiencing what stress does to your body” (*n* = 6); “The body says stop” (*n* = 51); “Now I will have to put myself as number 1 priority” (*n* = 53); “It is a learning process” (*n* = 84); “Trainer's specific suggestions what participants could feel” (*n* = 294).

**Figure 1:**
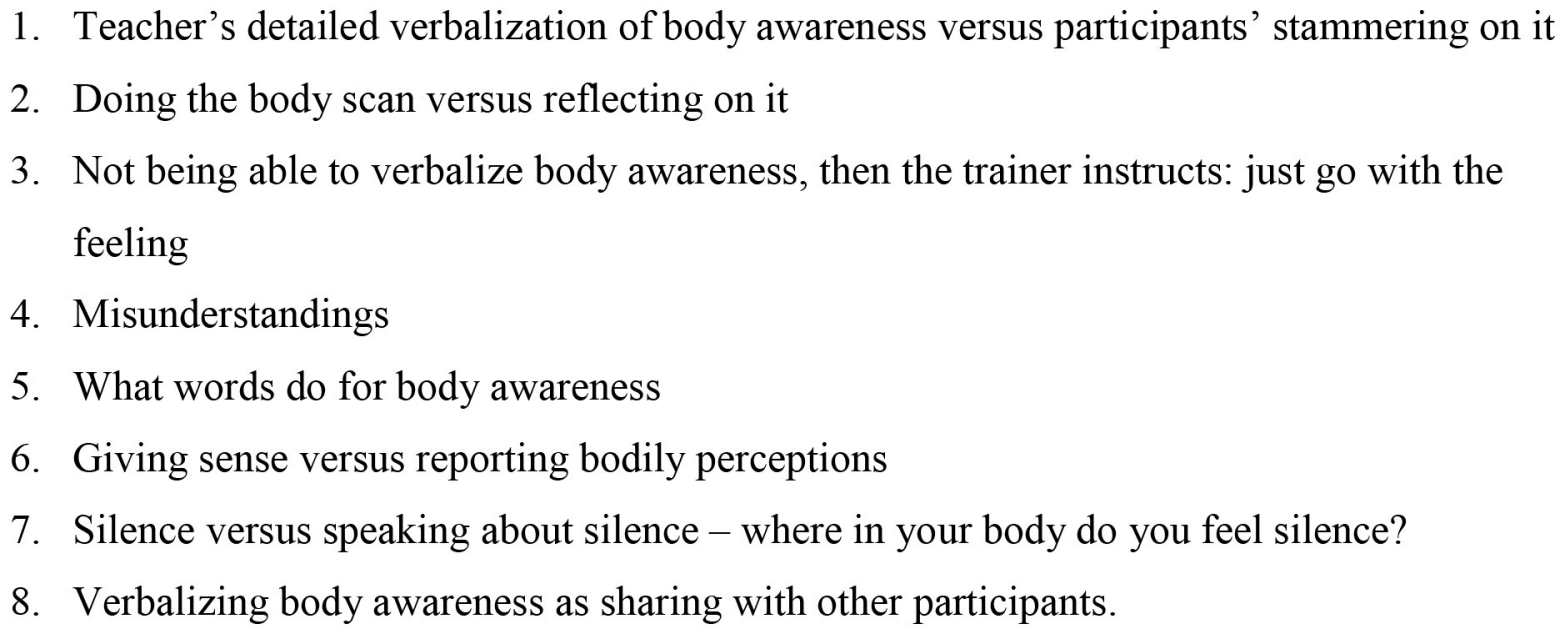
Overarching themes: verbalizing body awareness in transcripts of MBSR sessions.

The second phase of our analysis resulted in 8 overarching themes. We listed them in Figure 1.

The third phase of our analysis resulted in two main themes. First, the diverse ways participants verbalized their body awareness and how they were instructed or corrected in this by the trainer, and second, how verbalizing body awareness affected one's body and body awareness as such. Formulated in Wittgenstein's and Austin's vocabulary: (1) Shifting to the language-game of sense perception, and (2) Words acting on the body. In this section, we organized the presentation of our data according to these two main themes.

### 4.2 Verbalizing body awareness: shifting to the language-game of sense perception

One of the trainer's core interventions in the learning process of enhancing one's body awareness during MBSR sessions was asking: “How does it feel?” or “What does it do to you?” It was salient in our data that participants were guided by the trainer to answer these questions by reporting what they felt in or on their bodies. We will show this in detail in the following subsection. We called this “the language-game of *reporting sense perception*.”

It was also salient in our data that participants were inclined to play various language-games. Language-games such as: making rational judgments, reporting emotions, reporting felt inclinations, making sense of a situation by putting things into perspective, or looking for causes. We will provide examples of these in the following subsections.

#### 4.2.1 Rational judgments

In the first training session, the trainer invited the participants to introduce themselves. Clark did this by telling about problems in his family life, sharing that these problems caused stress-related health problems. He ended his introduction, saying: “Yes, then it is too much for me, and then your body says ‘stop.' Then it just stops.” After this, the following dialogue unfolds:

Trainer: Where did your body say stop? What, what
Clark: (*laughs*) Where? The location? Near to Paris.
Trainer: No, no, no, no, no, no, no, no. No, not where. But
where, where, where, where, where, what did not function?
Clark: Well, I erm, I erm, I erm, erm, erm, erm. I, I am, erm.
I had to stay in bed.

Clark did not answer the question “Where did your body say stop?” with reporting bodily experience—what the trainer expected him to do. Instead, he talked about geography, circumstances, and consequences. Clark did not understand that the trainer's question referred to sense perception. The trainer did not succeed in guiding Clark's language-game of making *rational judgments* to the language-game of *reporting sense perception*.

#### 4.2.2 Emotions

Participants were inclined to answer the questions “How does it feel?” or “What does it do to you?” by reporting an *emotion*. For instance, in the second training session, the trainer explored participants' experiences with the body scan directly after performing it. Giselle said that she was “fed up with herself” because she had so many distracting thoughts, but she was not able to tell the trainer what she felt in her body while being fed up.

In the next excerpt from the fifth training session, the trainer asked the participants to talk about their experiences with the homework for this fifth session: describing a stressful event that happened that week in their lives. Hannah answered that a close friend phoned her to tell sad news. Hannah told what happened during this phone call:

Hannah: But then I felt my whole body trembling,
everywhere, while I was talking to her on the phone, just
because of the tension. And, erm. So I found that very
distinctive, because normally I do feel it, with regard
to emotions, but not so much in my body, so now that was
very clear. And at the same time, I really thought it was very
stressful for myself. […]
Trainer**:** But you do feel now how those emotions, you are
very much aware now how
Hannah**:** Yes.
Trainer: they translate themselves in your body.

In the excerpt above, we see that Hannah became aware of what she felt in her body whilst having emotions and learned to describe those bodily feelings. Hannah learned the language-game of reporting sense perception, which was completely new to her.

Learning the language-game of reporting sense-perception, instead of reporting the feeling of emotions, can also be done while a dialogue between the trainer and a participant takes place. For instance, in the following dialogue, in the second training session, after the trainer had guided the participants through a meditation exercise in which stress had been induced. This stress was induced by making the participants imagine a stressful event—“someone familiar walking on the other side of the street who does not return your greeting”:

Giselle: To me, it felt a bit like a rejection or something.
Trainer: It felt like a rejection. […]
Giselle: Ouch. Yes.
Trainer: Like ouch.
Giselle: Yes.
Trainer: And may I ask, that ‘ouch', does it enter somewhere
in your body?
Giselle: Yes, in my stomach.
Trainer: In your stomach.
Giselle: Yes. […]
Trainer: And what do you feel there?
Giselle: Erm, a, a, a sort of tension, a sort of tickle, or a,
something like that.
Trainer: Okay. Tension, a tickle, something like that. Okay.

Thus, the trainer did not rest till Giselle reported not only that she felt a painful feeling of rejection, but also a localized sensation in her body.

#### 4.2.3 Felt inclinations

Some participants answered the question “How does it feel?” by reporting an inclination to do something. For instance, Iris reported that she felt like crying. We see that the trainer wants to know “where it gets under her skin.” The dialogue starts after a guided meditation to induce stress in the sixth training session—“Imagine you had an argument with a colleague, and then you want to talk with another colleague who says that she has no time”:

Iris: I felt, erm, yes, actually, it felt as if I was almost going to
cry. […]
Trainer: Okay. What did it do to you? […]
Iris: In my mind, I was already running to the bathroom to
have a good cry.
Trainer: So you were touched by it.
Iris: Yes, yes, I was surely touched by it, yes.
Trainer: Were you able to feel something? Where were you
touched by it? In the head? Memory? Or, or did it get
under your skin somewhere?
Iris: Well, yes, a bit, erm, a bit here, so to speak. [Iris pointed
at her throat.]
Trainer: At the top.
Iris: Yes.
Trainer: And what was it that you were feeling there?
Iris: Yes, a bit of tightness.
Trainer: Tightness.
Iris: Yes.
Trainer: A feeling of tightness at the top. Not nice.
Iris: No, because I do not want to cry at work, so then I very
much try to prevent that from happening.
Trainer: Yes, yes.
Iris: So, erm, then you are intensely fighting against
your emotion.

Here we see the same pattern as before: the participant used a specific language-game, and the trainer stimulated her to participate in the game of reporting sense perception. Iris reported that she felt like crying, she saw herself running to the bathroom, and reported that she was trying to prevent herself from crying. On the trainer's invitation, Iris switched to the language-game of sense perception and described an oppressed feeling in the region of her throat.

#### 4.2.4 Putting things into perspective

In the following excerpt, Iris described that she was sitting in her car, stressed, knowing she would be late for MBSR. She tried to deal with it by putting it into perspective, and the other participants encouraged her. The trainer then stopped this collective language-game and changed it to the language-game of reporting sense perception. This conversation took place in the sixth training session, when the trainer asked for experiences with the meditation exercises, and participants said that their experience with being in a rush had changed. Then the trainer asked Iris: “May I ask you, how did you sit in your car this morning?”

Iris: Well, I thought, yes, indeed, I tried to, my breathing,
erm, well, I thought, my car was stopped anyway, so I could
not go anywhere, and so I think okay, just try to relax. That
car is not moving any faster when I get stressed.
Trainer: And that is localized here. That is inside the head.
Iris: Yes.
Trainer: But was it also localized somewhere in your body,
that you were able to give in to “okay, then it is like this”?
Iris: Yes, I do find that hard to do. But anyway.
Doris: What is the worst thing that could happen if you
arrive five minutes late?
Iris: Yes. No. You are right.
Doris: The worst thing that could happen?
Iris: Well, I find it very annoying to be late, and when I see at
my navigation that I, then, that I will definitely arrive late,
then I think, oh yeah.
Then I am really fed up with it.
Julia: Good heavens.
Iris: Well, anyway, it is not the end of the world if I arrive
here three minutes late, but.
Doris: I assure you, nothing will happen.
Trainer: Well, I, I, I would
Hannah: Just try once then.
Julia: No, for myself. I think it is very annoying for yourself.
Trainer: But, interesting question, isn't it? Is there anything
you can maybe imagine that is much worse than arriving
three minutes late? But, but, then we go up here again, then
we go thinking, and that, that, well, that is fine, and it can be
helpful, once in a while, to reflect a bit on how things do
matter or not, and that can be of help. But, returning to this
context, then it is, primarily, go feeling how it feels.

Thus, we see that Iris found a way to reduce stress while sitting in her car. However, according to the trainer's intervention, stress reduction in mindfulness training is not to be reached by a psychological action like putting things into perspective. The trainer told Iris that it is about feeling how it feels, sitting stressed in her car. Maybe while feeling how it feels, the tension will go away by itself. Then Iris will find herself holding the wheel less tense, and will notice that her shoulders have dropped, just by first feeling her shrugged shoulders, as the trainer suggested to Iris. We see that the trainer does not deny that putting things into perspective may help, but “in this context” it is about “feeling how it feels.”

#### 4.2.5 Not finding the words

In the process of finding words for body awareness in MBSR, we saw that participants gradually learned to play the proper language-game of reporting sense perception. In the fourth MBSR session, the trainer asked the participants, directly after walking meditation, what they noticed in the way they were standing. Brandon answered that he felt himself standing “more solid on the ground,” and used the metaphor that he felt more “grounded.” Clark answered that he felt “less wobble,” and Hannah reported “warmth inside the feet.” But Lucy reported that she could not give words to the combined movement of both feet while walking slowly. We can discern another language-game here: reporting *not knowing, not finding the words*.

When the trainer stimulated participants to play the language-game of reporting sense perception, the dialogue often ended in “I don't know,” “I couldn't tell,” or “I don't remember.” We could say that *not knowing* is an often-played language-game in MBSR. Based on the trainer's reaction, we conclude that he preferred participants saying “I don't know” instead of “I think.” Reporting “I think” was directly replied with: “That is thinking, go feeling,” whereas reporting not knowing was followed by an invitation, such as: “Go discover it for yourself.” Not finding the words for body awareness implies, according to the trainer, that one has to go on practicing to feel one's body. Looking for the words then becomes a form of reflection, in which one becomes aware of the value of practicing.

### 4.3 Words acting on the body—Various speech acts in MBSR

#### 4.3.1 The trainer's words merely suggest what to feel and intend to help participants feel

At several moments in MBSR, the trainer explicitly explained what he was doing with words when giving verbal instructions during the body scan and other exercises for enhancing one's body awareness: “I will give some suggestions. Not that, not that you have to feel all that, but maybe it will help you to feel something” (quote from first training session). Using Austin's concepts, we can say that the *illocutionary act*, what the trainer is doing here with words, is *suggesting*. We can also say that what he is doing here is *trying to help*. The *perlocutionary act*, what is done to the listener by means of the spoken words, whether those words actually helped the participants, is hereby not given. The trainer acknowledged that his suggestions may or may not be helpful. One of the suggestions the trainer made a lot was to guide the movement of inspiration and expiration with words like “space” and “loose.” Some participants found those words helpful, including Hannah. She said in the third training session, after finishing the exercise of the body scan in a sitting position:

Hannah: I found, I feel also a change, when you called it
space and loose.
Trainer: Okay. What did it do then?
Hannah: Then I thought, yes, it was nice, it just belonged to
the breathing, or something.
Trainer: Nice words.
Hannah: Then I really thought like, oh, and then it did go
a bit more natural, or a bit more loose, I think, in the
end. Me myself, I am often still very tightened.
Trainer: Okay.
Hannah: So, then I thought, well, it does help what words
you use, or which words you do not use.

Thus, we saw testimonies of the trainer's words as *perlocutionary acts*: participants said that they were actually affected by his words, and that their respiration changed. In our study, the trainer's *illocutionary act* of trying to help did not result for all participants in the *perlocutionary act* of actually helping the participant. For instance, one of the trainer's suggestions about breathing, “breathing toward one's legs,” during the body scan in the lying position in the first training session, inhibited participant Brandon from breathing: “I was no longer able to breathe out, actually.” The trainer's words constituted the *perlocutionary act* of blocking the breathing.

Not only the trainer's words but also the words of instruction audios, used as homework, can be seen as perlocutionary acts if these words affect the listener. Giselle told, in the seventh training session, about her experience with a homework audio that contained the metaphoric mountain meditation. In this meditation, participants have to imagine that they are a mountain that remains the same inside, while the weather circumstances are changing. Giselle: “I really liked it very much, I felt, I felt literally, that I was rooted to the ground.”

#### 4.3.2 Locutionary act—The trainer's voice vs. instruction audiotapes

Additionally, the *locutionary* act, the sounds uttered by the trainer or produced by the instructional audios, played a role in the learning and teaching process in MBSR. Participants differed in how they preferred to hear the instruction audios: putting it on speakers, streaming it to their televisions, or using headphones in order to block other sounds. Participants also differed in how they appreciated the tone of voice of these locutionary acts; some liked the voice, others did not.

The trainer emphasized that participants should just notice and feel how they experience the various voices, the various locutionary acts, whether they like them or not. The following excerpt comes from the fourth training session, when the participants were evaluating the body scan they had done in a lying position, guided by the trainer:

Clark: When I hear your voice then I hear, very nice. Good,
just precisely the right resonance.
Trainer: That is nice. That is nice that you have this
experience. I do realize that a calm voice matters. That
resonance matters. But it is just the art to see what it does to
you, how it feels, with maybe an irritating voice, or a voice
that makes mistakes. Just go feeling. Where do you feel that
in your body?

#### 4.3.3 Doing away with the words

In the seventh training session, the trainer told the participants that eventually it is not about his doing things with words, but about the participants doing things themselves:

Trainer: It is not me who is doing that, it is you. You are
doing that all by yourself. Me or some audio instruction
guides you in this, but you, you take the time, you allow
yourself the time to lie down like this, to learn to let go,
and then to see, hey, this is still here.

In the fourth training session, the trainer instructed the participants to do their exercises at home. However, Julia responded that she found it difficult to do exercises without being guided by a voice. The trainer told Julia that that is just part of the learning process. In the beginning, it may well be easier to do exercises while listening to an instructing voice. However, as the trainer promised, eventually Julia will be able to do the exercises on her own, and she will not need the instructor's voice anymore.

## 5 Discussion

### 5.1 Reflection on results

In this study, we analyzed how language is used in the training practice of enhancing one's body awareness in MBSR, based on a participant observation study. Our first research question was how participants verbalize their experiences of body awareness in an 8-week mindfulness training. We found in our study that verbalizing body awareness was difficult for participants. It was hard for them to give words to the bodily sensations they felt in and on their bodies. When asked to verbalize their body awareness, participants talked about feeling emotions, rationalized about causes, or put what happened into perspective, instead of reporting bodily sensations. For instance, we analyzed that the expression used, “My body said stop,” was a rational judgment that bypassed what can actually be felt in one's body. Additionally, “putting things into perspective” is a form of cognitive reappraisal, a psychological strategy for emotion regulation that taxes cognitive resources (Wenzel et al., 2023). When participants talked about feeling emotions, they found it hard to put into words what they actually felt in their bodies. Instead, they only mentioned the name of an emotion, for instance, “being fed up.”

Our second research question was how MBSR participants were stimulated by the trainer to give words to their bodily experiences. We found that the trainer stimulated the participants to talk about their bodily feelings, what they felt in and on their bodies. It was salient in our data that when the trainer asked the participants how they felt, he stimulated them to give an answer that was related to their bodily feelings. This was not an answer that was related to the feeling of an emotion. Nor was it an answer related to a “felt inclination” (Stout, 2022, p. 286), for instance, being inclined to run to the bathroom to cry, nor to rational thoughts, nor to judgments. According to our results, the kind of feelings that the trainer stimulated the participants to report as an answer to the question “What do you feel?” were interoceptive and proprioceptive feelings. These are, for instance, feeling warmth, feeling one's heartbeat, feeling the movement of one's chest, feeling a tickle in one's belly, feeling the tension in one's muscles, or feeling the position of one's joints (Leder, 1990; Mehling et al., 2009; Meijsing, 2022). Our results on the trainer's preference on how to report about one's experiences with body awareness align with Mehling et al.'s (2009) definition of “body awareness.” These authors define body awareness as “the subjective, phenomenological aspect of proprioception and interoception that enters conscious awareness” (2).

Thus, we saw in our data that MBSR participants were inclined to answer the question “How does it feel?” in many different ways. However, these were all different from the way the trainer encouraged the participants to verbalize what they felt. We reflected on this through the lens of our theoretical framework. “How does it feel?” is a question that is open to multiple interpretations. The answer “I feel angry” is, obviously, no less adequate than “I feel my heart beating.” It is just another interpretation of “feeling.” In Wittgenstein's (1953/2009) vocabulary, we can say that these different uses of “feeling” are different language-games. We concluded that in our data, the language-game that was instructed by the MBSR-trainer in this particular MBSR training is about reporting bodily feelings. We named this “the language-game of reporting sense perceptions.” We can understand the process of enhancing one's body awareness in MBSR as the process of learning the language-game of reporting sense perception. Mindfulness is about being aware in the present moment and cultivating a non-judging attitude (Santorelli and Kabat-Zinn, 2014; Kabat-Zinn, 2020, 21). Therefore, we can understand enhancing one's body awareness in MBSR—learning the language-game of reporting sense perception—also as learning the language-game of reporting what one can actually be aware of. This is reporting what one can actually feel here and now in and on one's own body, instead of jumping to judgments or conclusions whilst bypassing what can be felt.

It is important to note that in this study, we explored and did not prescribe how words are used to verbalize body awareness in one particular MBSR course. However, we concluded from our findings that there is a prescriptive element involved in this MBSR training about how participants should verbalize their body awareness. We described this prescriptive element by making use of the concept of language-games. One could say that in this prescriptive element, the trainer's authority emerges. We see the same trainer authority already in Kabat-Zinn's (1982) early study on mindfulness practice. In his study, the learning process of becoming aware of the difference between feeling emotions and feeling bodily sensations, which we found in our study, was already mentioned. In this early study on mindfulness practice, chronic pain patients learned to separate the feeling of bodily pain signals from the emotional part of the pain experience. In mindfulness vocabulary, we could say: participants learned to cultivate a non-judging attitude (Santorelli and Kabat-Zinn, 2014; Kabat-Zinn, 2020, p. 21). The findings in our study align with current insights on emotion regulation therapy. The first step in these therapies is not recognizing one's emotions, nor looking for causes or solutions: the first step is learning to feel, learning to become aware of bodily sensations (Price and Hooven, 2018). As we found in our study, in mindfulness practice, feeling bodily sensations is practiced by directing one's awareness to one's body.

In our data, participants who were not able to express what they felt in and on their bodies were stimulated by the trainer to say that they did not know. Instead of saying “I think.” This privileged position of not knowing over theorizing is reflected in the notion of the “beginner's mind”—one of the attitudes cultivated in mindfulness practice (Kabat-Zinn, 2020, p. 24). In Zen meditation, consistent with mindfulness practice, a practice can be found of responding “I don't know” to every thought that comes up (Fronsdal, 2004). MBSR participants were instructed to *feel* their body instead of *thinking* about it. Others have criticized this emphasis on feeling instead of thinking: Sauerborn et al. (2022) argue that in mindfulness interventions, an idealized body is constructed, which handles emotions in an allegedly authentic, bodily, non-cognitive way. They discern in mindfulness's imperative to just feel your body without thinking about it, a paradox of ‘glorification of authenticity and manipulation of emotions' (1,055). The paradox they discern is that this prescription of “feeling without thinking” is commodified as a means to control your feelings and emotions. Moreover, we can discern another paradox here, which Merleau-Ponty (2012, p. 408) described as the paradox of expression. When we speak, we make use of existing words, trying to express something new that was not expressed in words before. There is thinking involved in expressing what one feels. Therefore, it is paradoxical to speak about the pre-reflective body, the body that is felt without thinking about it. One of us elaborated on this paradox of expression in relation to mindfulness practice elsewhere (Van den Bold, 2023).

Our third research question was how language was used by the MBSR trainer to enhance participants' body awareness. We analyzed this language use, drawing on Austin's (1962) concept of speech acts. Based on our data, we analyzed that the trainer's detailed verbal descriptions of what participants could feel in and on their bodies were intended to help participants feel. The trainer's verbal descriptions were intended to help participants enhance their body awareness. This is the illocutionary act, what the trainer intended to do with his words. The perlocutionary act, which is what is actually achieved by the words spoken, differed per person and per situation. We saw in our data that the trainer's suggestion on what to feel, according to the participants, sometimes helped them to feel, and sometimes not. We also found that Austin's concept of the locutionary act, the way the words were actually uttered, was important for the participants.

### 5.2 New insights

Summing up, our results extend existing scholarship, as we not only found that learning the practice is learning the language, as Mathews and Anderson (2021) did. We described which language-game was considered to be a *proper* one for enhancing one's body awareness in MBSR. Moreover, we also described and analyzed the language-games that participants were inclined to play instead of reporting sense perceptions. This detailed insight into language-games in MBSR is new. Our findings also extend existing scholarship regarding the application of Austin's theory of speech acts. Others described the performativity of words on participants' body awareness, based on interviews (Petitmengin et al., 2017). In our study, however, we analyzed the *actual linguistic practices* in an MBSR training course, including the verbal interactions between the trainer and the participants. Moreover, not only did we describe and analyze the performativity of speech acts in MBSR, but we also described and analyzed all three sorts of speech acts within Austin's concept: the locutionary act, the illocutionary act, and the perlocutionary act. By doing so, this study showed extensively how enhancing one's body awareness is done with words in MBSR.

### 5.3 Strengths and limitations

A strength of this study is that it analyzed what is said live by the participants and by the trainer in an MBSR training course. This method, opposed to *post-hoc* reflection and as opposed to the analysis of meditation apps, revealed how language was actually used during an MBSR course. Another strength of our study is that we analyzed all eight complete sessions of the MBSR training. Moreover, we analyzed our data through the lens of Wittgenstein's and Austin's philosophies, which has rarely been done before.

Our study also has limitations. This participant observation study was analyzed in a qualitative way, guided by a philosophy of language approach. As such, our method can be considered more subjective than quantitative linguistic analyses, such as counting occurrences of specific words or analyzing patterns of turn-taking in conversations. It is in the nature of qualitative studies that their outcomes are not generalizable. Moreover, our study consisted of the analysis of just one particular MBSR course. Because of that, we investigated only the language use of one particular trainer and of a small culturally homogenous group of participants. Therefore, our results cannot be extrapolated directly to similar MBSR courses in different demographic contexts, including participants and trainers with different socio-economic and ethnic backgrounds. A suggestion for further research would be to see whether our philosophical analysis holds for other MBSR courses led by other trainers. It would be interesting to see what differences and invariants could be detected between the various trainers in their use of language, seen through a philosophical lens of language-games. Our study design did not include triangulation. However, it would have been interesting to see how the trainer and participants would have responded to our study results.

Another limitation of our study is that we did not collect individual background characteristics. This was a deliberate decision, not to interfere with the training's natural course. However, it turned out to be a drawback, as language use is related to socioeconomic status and to education level.

In this study, we applied philosophical categories to live dialogue. These philosophical theories provided us with a lens to look at our data. However, using these theories may also have a drawback. These philosophical theories may not only have functioned as a lens to look at the data, but may also have guided our interpretation in a certain direction.

### 5.4 Conclusion and implications

Our results contribute to the debate on the relation between language and body awareness. They corroborate both the view of language as an obstacle to body awareness and the contradictory view of language being helpful for body awareness. We showed that the relation between language and body awareness is ambiguous, and that it differed per participant and per situation. Our results contradicted a third view that body awareness cannot be expressed in words, as it is considered non-conceptual. We can admit that it is paradoxical to speak about the pre-reflective body, the body that is felt without thinking about it. However, when we looked at how enhancing one's body awareness in MBSR is done, and how language is used in this practice, we saw that participants learn to master a proper language-game. We suggested that the language-game of reporting sense perception—instead of reporting emotions or reporting causes—is a way MBSR participants learn to speak about being aware of their bodies. As our findings are based on a single case study, one particular MBSR training with one particular trainer, we recommend replicating the study for other MBSR training groups and trainers, in diverse cultural groups and diverse languages. We also suggest focus group studies to explore how MBSR trainers receive the concept of language-games applied to trainer-participant dialogue.

We tentatively suggest what our findings may mean for mindfulness practice, as it is in the nature of our qualitative study method that we cannot generalize our findings. Audio tapes and apps with mindfulness instructions can provide the words acting on the body, but they cannot deliver personal feedback on the use of language-games. Listeners to these tapes will not be corrected when they stray into other language-games, for instance, on emotions or causes. Therein lies the trainer's added value. Someone present who will not rest, saying: “That is thinking, go feeling.” This conclusion has implications for the widespread use of mindfulness apps and for the popular use of recorded versions of the body scan. We suspect that these forms of practicing mindfulness might hinder the actual learning process of feeling one's own body. We hypothesize that the use of these apps, without the possibility of engaging in a living dialogue with a trainer, could involve the risk that users bypass their bodily experiences and go on rationalizing about their experiences instead of feeling them. This hypothesis requires further research.

Another implication of our study is that it may contribute to mindfulness pedagogy. Our study makes explicit that a dialogue in the form of expressing sense perceptions may contribute to participants' body awareness. The implication would be that this could be made more explicit in training materials. Another implication of our study would be that in training materials, the difference between reporting sense perceptions and reporting emotions could be clarified explicitly. Further research is warranted to see how the results of this study of MBSR language use may differ from language use in various cultural contexts.

## Data Availability

The data underlying this study cannot be made publicly available, for ethical reasons. These data, containing audio files and full transcript, cannot be made publicly available as this would compromise participant confidentiality and privacy. Requests to access the datasets should be directed to: I.F.D.vdnBold@tilburguniversity.edu.
